# Editorial: Advanced technologies for energy saving, plant quality control and mechanization development in plant factory

**DOI:** 10.3389/fpls.2023.1193158

**Published:** 2023-05-19

**Authors:** Yuxin Tong, Myung-Min Oh, Wei Fang

**Affiliations:** ^1^ Institute of Environment and Sustainable Development in Agriculture, Chinese Academy of Agricultural Sciences, Beijing, China; ^2^ Division of Animal, Horticultural and Food Sciences, Chungbuk National University, Cheongju, Republic of Korea; ^3^ Department of Biomechatronics Engineering, National Taiwan University, Taiwan, China

**Keywords:** computer vision, energy saving, plant quality control, mechanization development, plant factory

We are facing global problems of food shortage and/or unstable supply, resource (land, water, fossil fuel, etc.) shortages and environment degradation. To solve these problems, we need to employ plant production systems with less resource consumption and environmental degradation for high yield and quality plant production. Plant factories with artificial light (PFALs) with the benefits of 1) high input resources use efficiencies; 2) clean, safe and nutrient-rich plant production without pesticides; 3) vertical cultivation with high plant density and annual productivity; and 4) attracting young laborers due to the comfortable and labor-saving working environment, can be considered as one of the above systems. However, some major problems, such as the high initial investment and running cost, still need to be solved in PFALs. To address and update our knowledge on “*Advanced Technologies for Energy Saving, Plant Quality Control and Mechanization Development in PFAL*,” we organized this Research Topic.

## Plant quality control

Plant quality can be managed by environmental factors control. CO_2_ concentration exerts a substrantial impact on crop yield and quality as the source of carbon, while frequent CO_2_ deficiency in PFALs during the daytime limits the improvement of crop productivity. Wang et al. reviewed the current situation and research progress on CO_2_ enrichment systems, including characteristics, limitations, and control strategies. Plants have different morphological and physiological responses to specific light qualities and many plant species are more sensitive to red and blue lights. Li et al. aimed to evaluate the response of leaf photosynthesis, biomass accumulation, and growth of pepper seedlings under various proportions of mixed red and blue lights. The results indicated that the red-blue ratio of 3:1 was effective in improving photomorphogenesis and photosynthesis of sweet pepper plants, achieving higher biomass accumulation and energy utilization efficiency simultaneously. Zhang et al. conducted a study to clarify the effect of different spectral combinations on the growth, yield and nutritional quality of pea sprouts under a long photoperiod (22h light/2h dark). The red-blue ratio of 2:1 was suggested to be the suitable light quality combination for improving the growth and quality of pea sprouts under a long photoperiod. Li et al. investigated different light spectra on growth, nutritional quality and antioxidant property of winter wheat seedlings in a PFAL. The authors found that red light promoted carbohydrate accumulation in wheat seedlings, while blue light promoted the antioxidant level of wheat seedlings. The above studies can be helpful for light spectral formulating to improve plant growth, quality, and light use efficiency in PFALs. Lee and Goto explored the short-term effects of ozone exposure on the growth and accumulation of bioactive compounds in red lettuce leaves grown in a PFAL. The authors presented the efficiency and advantages of using ozone as an elicitor for bioactive compound accumulation in red leaf lettuce seedlings. Wittayathanarattana et al. presented a study to establish the short-term cooling root-zone temperature under controlled environments that could improve antioxidant capacity and bioactive compound accumulation in amaranth (*Amaranthus tricolor* L.) baby leaf without causing an abnormal appearance.

## Computer vision and plant phenotype

Accurate and timely crop growth monitoring in PFALs is essential for management decision-making. Lin et al. proposed a multi-modal fusion-based deep learning model for automatically monitoring lettuce shoot fresh weight by utilizing RGB-D images. The model improved the fresh weight estimation performance by simultaneously leveraging the advantages of empirical geometric traits and deep neural features. The study suggests that the combination of multi-modal data fusion and deep neural and empirical geometric features is a promising approach for estimating fresh weight. Yoon et al. developed a model for predicting phenolic accumulation in kale (*Brassica oleracea* L. var. *acephala*) according to UV-B radiation interception and growth stage. The spatial distribution of UV-B radiation interception in plants was quantified using a ray-tracing simulation with 3D-scanned plant models. Plants adapt their photosynthetic and photoprotective pigment content to the surrounding environment, which means that changes in leaf biochemistry can be used as markers for detecting enhancements in plant nutritional quality and early warning signs of stress in PFALs, contributing to making production more efficient and precise. Cammarisano et al. proposed the use of leaf spectroscopy and mathematical modeling for non-invasive estimation of leaf pigments to monitor of plant cultivation. Ratios of pigments such as chlorophylls/carotenoids and anthocyanins/chlorophylls have demonstrated potential for indicating changes in plant stress status and nutritional quality enhancements.

## Economic feasibility

Despite high expectations, the diffusion of commercial crop production in PFALs has been slowed down worldwide due to the significant initial investment and running costs. Uyeh et al. provided a decision tool that could facilitate improved decision-making in retrofitting PFALs and help efficiently facilitate mechanization and automation. The authors considered minimizing the total cost for retrofitting and maximizing the yearly net profit as two objectives and showed the usefulness of this methodology/software to compare the cost performance of different plant production systems. Zhuang et al. estimated the degree of the scale economies and found that lettuce is a well-established PFAL crop, but strawberry is not. The scale of PFALs is an important factor in determining the economic performance of PFALs’ crop production. They also found that PFALs’ crop production is highly sensitive, with a 30% decline in the lettuce price bring PFALs to bankruptcy, and a 20% increase in strawberries yield per unit transforming it into an economically viable PFAL crop. The results of their research can help access the economic feasibility of PFALs’ crop production. Lanoue et al. discovered an innovative lighting strategy that uses 24 hours of low- intensity lighting to provide the desired amount of light for plant growth. This lighting strategy reduces capital costs for light fixtures and electricity used for air conditioning since low-intensity lighting requires fewer light fixtures and reduces heat load from lighting and plant transpiration. The strategy increased yield and nutritional contents while reducing the electricity cost per unit of product, significantly improving the sustainability of microgreen production with PFALs.

## Author contributions

YT drafted the manuscript. All the authors contributed to the article and approved the submitted version.

